# The prophylactic anti-aging effect of aspirin (acetylsalicylic acid) on oxidative stress-induced damage in the buccal mucosa of D-galactose-induced aged rats

**DOI:** 10.1038/s41598-025-94566-1

**Published:** 2025-04-16

**Authors:** Mohamed Khaled Mohamed Maria, Maha Hassan Bashir, Amira E. Fares, Nermeen AbuBakr

**Affiliations:** 1 Oral Biology Department, Faculty of Dentistry, Cairo University, Cairo, Egypt; 2 Oral Biology Department, Faculty of Dentistry, Suez University, Suez, Egypt

**Keywords:** Aspirin, Antiaging, Buccal mucosa, Comet assay, iNOS, Senescence, Preventive medicine, Experimental models of disease, Preclinical research, Geriatrics

## Abstract

Most living organisms experience time-dependent functional deterioration as they age. To combat aging, aspirin was proposed as an already well-studied drug. However, its antiaging effect is neither well studied nor understood. So, this study intended to assess the proposed antiaging effect of aspirin. Three groups of seven adult male albino rats were established. The control group received saline, the aging model group got a daily single D-galactose subcutaneous injection (300 mg/kg), and the aspirin group consisted of D-galactose-induced aged rats that received a daily aspirin oral dose (60 mg/kg). Drugs were given for 8 weeks. Then, malondialdehyde (MDA) blood level was evaluated, and rats were euthanized. Buccal mucosa samples were obtained for inducible nitric oxide synthase (iNOS) gene expression, histopathological, ultrastructural, and comet analyses. MDA blood level, iNOS gene expression and DNA damage examined by comet assay displayed a significant reduction in the aspirin group when compared to the aging model group. Histopathological and ultrastructural results showed that aspirin ameliorated most of the degenerative signs caused by D-galactose. Thus, it was deduced that aspirin had promising results as an antiaging pharmaceutical agent. However, more studies are needed regarding its translation to human trials.

## Introduction

Aging can be broadly defined as the functional deterioration and physiological integrity loss that occurs throughout time and affects most living organisms^1^. Aging can be caused by many factors, including genetic problems, environmental factors, diseases, or finally the inherent aging process that occurs even if optimal living conditions are achieved^2^.

Aging has some specific characteristics, including cellular and tissue changes, reduction in physiological capacity, reduced adaptation to environmental stimuli, and increased vulnerability to diseases like diabetes, cancer, heart problems, and neurological disorders. All these characteristics lead to increased mortality chances^1,3^.

Antiaging strategies utilizing medication administration and drug combinations continue to be appealing. Recent antiaging research is mainly focused on pharmaceutical agents that are well-known and accepted by the Food and Drug Administration (FDA), like statins, metformin, rapamycin, and aspirin^4^.

Aspirin (acetylsalicylic acid) is one of the most famous pharmaceutical agents. It has well-investigated properties regarding its analgesic, antipyretic, and anti-inflammatory effects. It aids in preventing and ameliorating cardiovascular diseases and enhancing cognitive function^4,5^. Aspirin also has other advantages, like its low cost and simplicity of treatment, as it is mainly ingested orally^4^.

Repurposing of aspirin usage as an antiaging pharmaceutical agent was suggested due to its previously mentioned advantages. However, its complementing benefits for age-related illnesses and antiaging properties are still under investigation.

To fill this gap of knowledge, this study was meant to investigate the effect of aspirin in ameliorating age-related alterations on the buccal mucosa of D-galactose-induced aged albino rats and to spot some light on the possible mechanisms that may lead to this amelioration by assessing malondialdehyde (MDA) blood level, inducible nitric oxide synthase (iNOS) gene expression using quantitative real-time polymerase chain reaction (qRT-PCR), histologically, ultrastructurally and by measuring the extent of DNA damage.

## Materials and methods

### Chemicals and reagents

D-galactose, in the form of powder, was purchased from El Gomhoureya for Drugs Trade & Medical Supplies, Egypt. Aspirin, in the form of tablets, was purchased from El-Ezaby pharmacy, Egypt.

### Ethical approval

The present work was conducted in accordance with the regulations and guidelines of the Institutional Animal Care and Use Committee at Cairo University. This study protocol was approved by the committee (approval number: CU/ III /F/ 40/ 20). The ARRIVE guidelines have been followed in this animal study.

### Experimental animals

In this study, 21 adult male albino rats (species: Rattus norvegicus), four months old were employed. They were randomly selected and housed in the animal house located in Faculty of Medicine, Cairo University. They were maintained inside clean cages, in a sterile and regulated setting at 20–24 ºC, provided with regular rat pellets, a 12:12 light-dark cycle, moderate humidity, and unrestricted availability of fresh tap water.

### Induction of aging

Aging was induced using a daily single subcutaneous D-galactose injection (300mg/kg/day) for 8 weeks. 0.9% normal saline was used to dissolve D-galactose^6^.

### Study design

Rats were split into three groups at random (*n* = 7): Group I was the normal control group; Group II was the aging model group; and Group III was the aspirin group. D-galactose was injected subcutaneously after being dissolved in 0.9% normal saline. Aspirin was dissolved in distilled water and administered via oral gavage. The normal control group was injected subcutaneously once a day with normal saline, while the other two groups were injected subcutaneously once a day with D-galactose (300 mg/kg/day) for eight weeks. Meanwhile, the aspirin group received aspirin (60 mg/kg/day)^7^ once a day via oral gavage for eight weeks, while the normal and aging model groups were given equal volumes of distilled water in the same manner.

### Biochemical analysis

After eight weeks, blood samples were randomly drawn from each group, and MDA blood level was evaluated to measure lipid peroxidation. The lipid peroxidation (MDA) colorimetric/fluorometric test kit (Biovision) was utilized in compliance with the manufacturer’s instructions.

### Animals’ sacrifice and tissue preparation

All rats were euthanized by using an overdose of ketamine and xylazine after 8 weeks from the beginning of the experiment^8^. Buccal mucosa opposing the upper first molar was dissected and processed for:

### Quantitative real-time polymerase chain reaction (qRT-PCR) for iNOS expression

After homogenizing the samples, the RNA Easy Mini Kit (Qiagen) was used to extract total RNA, and Beckman dual spectrophotometer (United States of America) was used to assess the amount and quality of the RNA. With a high-capacity cDNA Reverse Transcriptase kit from Applied Biosystem in the United States of America, 1000 ng of the total RNA from each sample were utilized for cDNA synthesis by reverse transcription. Then, using the Step One equipment (Applied Biosystem, United States of America), and Fermentas’ Syber Green I PCR Master Kit, the cDNA was amplified on a 48-well plate as followed: After 10 min of enzyme activation at 95 ^o^C, the amplification phase was conducted utilizing 40 cycles of 15 s at 95 ^o^C, 20 s at 55 ^o^C, and 30 s at 72 ^o^C^9^. BioRad syber green PCR MIX kit was used to check the amplified double strand gene in real time. Using the ΔΔCt approach, changes in the target gene (iNOS) expression were standardized in relation to the mean critical threshold values of the housekeeping gene, glyceraldehyde 3-phosphate dehydrogenase (GAPDH). One micrometer of each of the two target gene-specific primers was employed. Table (1) shows the primers’ sequence for each gene.

**Table 1:** Primers’ sequence

Target gene	Primer sequence: 5`- 3`	Gene accession number
**iNOS**	Forward: 5’-TCTGTGCTAATGCGGAAGGTCATG-3’ Reverse: 5’-TTGTCACCACCAGCAGTAGTTGTTC-3’	XM_220732
**GAPDH**	Forward: 5’-AGTGCCAGCCTCGTCTCATA-3’ Reverse: 5’-GATGGTGATGGGTTTCCCGT-3’	XM_216453

### Histopathological examination

After being dissected, the buccal mucosa samples were preserved in 10% neutral buffered formalin for a full day. The samples were incorporated in paraffin wax after going through a series of graded alcohol processes and xylene. Sections of 4 microns were fixed on glass slides and hematoxylin and eosin (H&E) stained. Light microscopy was used to analyze the histological sections.

### Ultrastructural examination

The samples were preserved in 2.5% glutaraldehyde in 0.1 M cacodylate buffer (pH 7.4). The same solution containing 1% potassium ferrocyanide and 1% osmium tetroxide was then used to post-fix them for an hour. After only 50 s of exposure to 0.15% tannic acid, the samples were shaken and incubated for one and a half hours in 1% uranyl acetate. The next step was to dehydrate the samples in ethanol and then embed in resin. A Reichert-Jung Ultra cut E ultramicrotome was used to cut semi-thin sections^10^. Transmission electron microscopy equipment (Philips XL 30) was used to investigate ultrathin slices doubly stained with uranyl acetate and lead citrate material.

### Comet assay

The comet test was carried out in an alkaline environment, essentially following the guidelines provided by Tice et al.^11^. A 0.5% typical agarose coating was applied first to ordinary microscope slides. Every slide was submerged in a lysing solution for one hour at 4 ◦C. The electrophoresis process was carried out at room temperature for 20 min at 300 mA and 25 V. The slides were put into an electrophoresis tank that contained a newly made alkaline buffer. Lastly, 60 µl of a 20 µl/ml ethidium bromide stain was applied to the DNA samples. A Leica fluorescence microscope and an automated image processing system were used to measure fifty randomly chosen keratinocytes for the comet test.

### Statistical analysis

A commercial software package (SPSS Chicago, version 26, USA) was used. The data showed a normal distribution according to the Kolmogorov-Smirnov normality test. The terms mean and standard deviation (SD) were used to describe numerical data. The data was compared using the one-way analysis of variance (ANOVA) test. A significant threshold of *P* < 0.05 was established. Every test included two tails. Pairwise comparisons were made using the Tukey post-hoc test if the ANOVA findings were statistically significant.

## Results

### Biochemical analysis

**Fig. 1:**
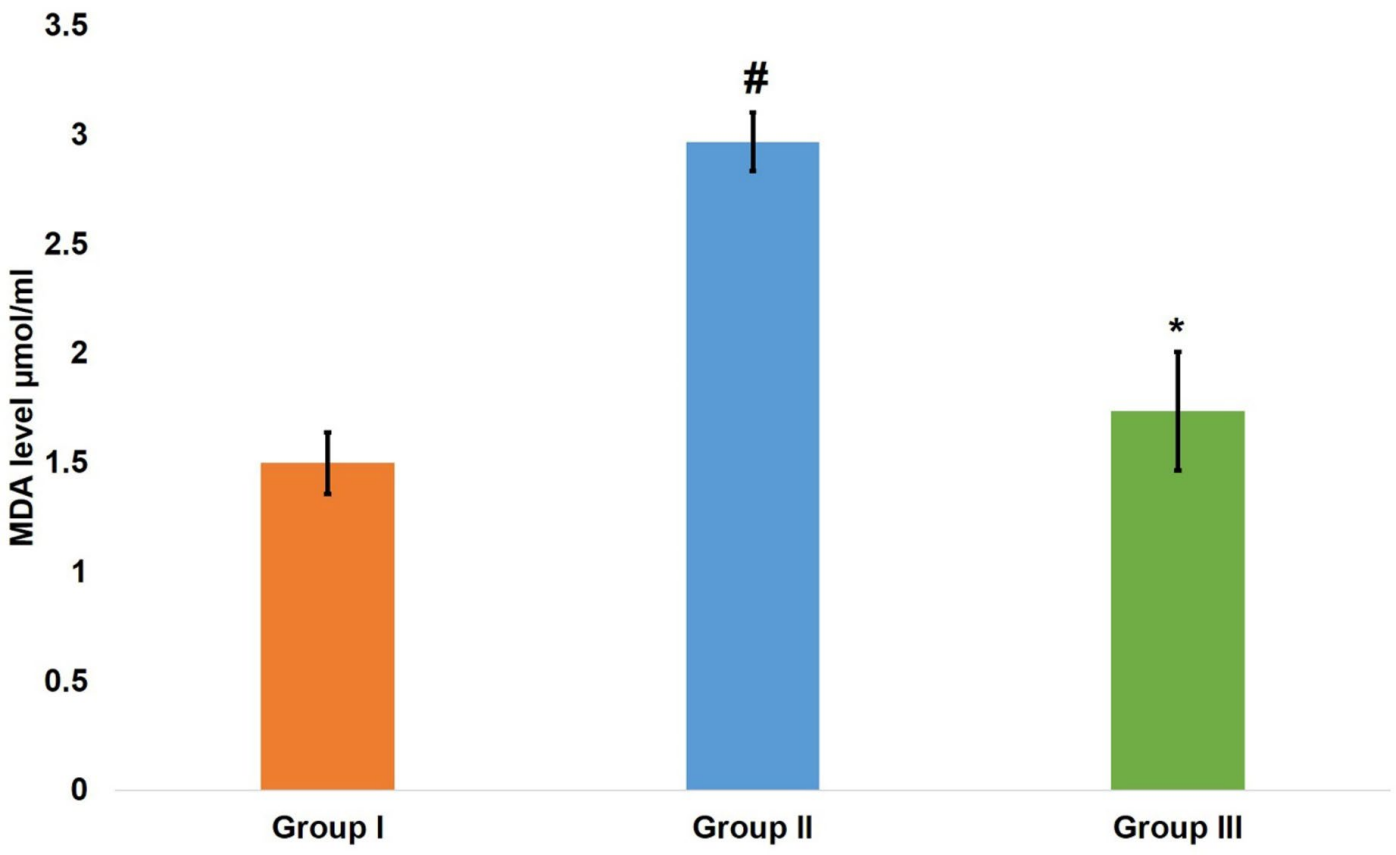
A bar chart representing mean ± SD of MDA level. # indicates significant difference versus control group. * indicates significant difference versus aging model group. One way ANOVA at p value < 0.05.

The results of a one-way ANOVA test showed a difference of statistical significance (*P* < 0.001) in MDA levels between all groups at 8 weeks. Moving to pairwise comparisons, the aging model group values were statistically significantly higher than both the control group (*P* < 0.001) and the aspirin group (*P* < 0.001). Nevertheless, there was no statistically significant difference between the aspirin and control groups (*P* = 0.082) (Fig. 1).

### iNOS gene expression analysis

**Fig. 2:**
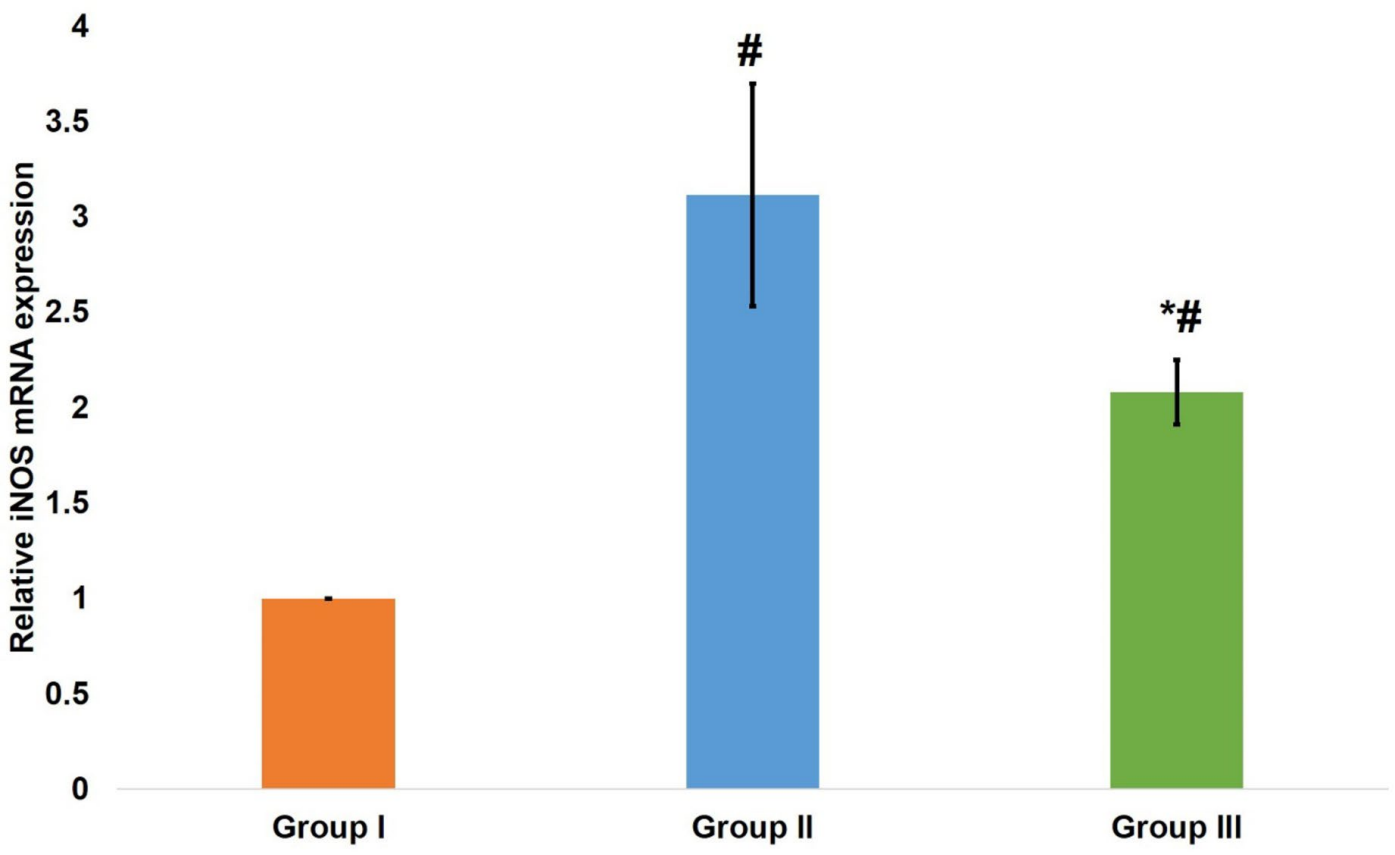
A bar chart representing mean ± SD of iNOS mRNA gene expression. # indicates significant difference versus control group. * indicates significant difference versus aging model group. One way ANOVA at p value < 0.05.

iNOS gene expression was analyzed in all groups. There was a significant variation (*P* < 0.001) between all groups using a one-way ANOVA test. As for the post-hoc pairwise comparisons between groups, the Tukey test revealed a significant difference between the control group and aging model group (*P* < 0.001), the control group and aspirin group (*P* < 0.001), and finally between the aging model group and aspirin group (*P* < 0.001) (Fig. 2).

### Histopathological results

**Fig. 3:**
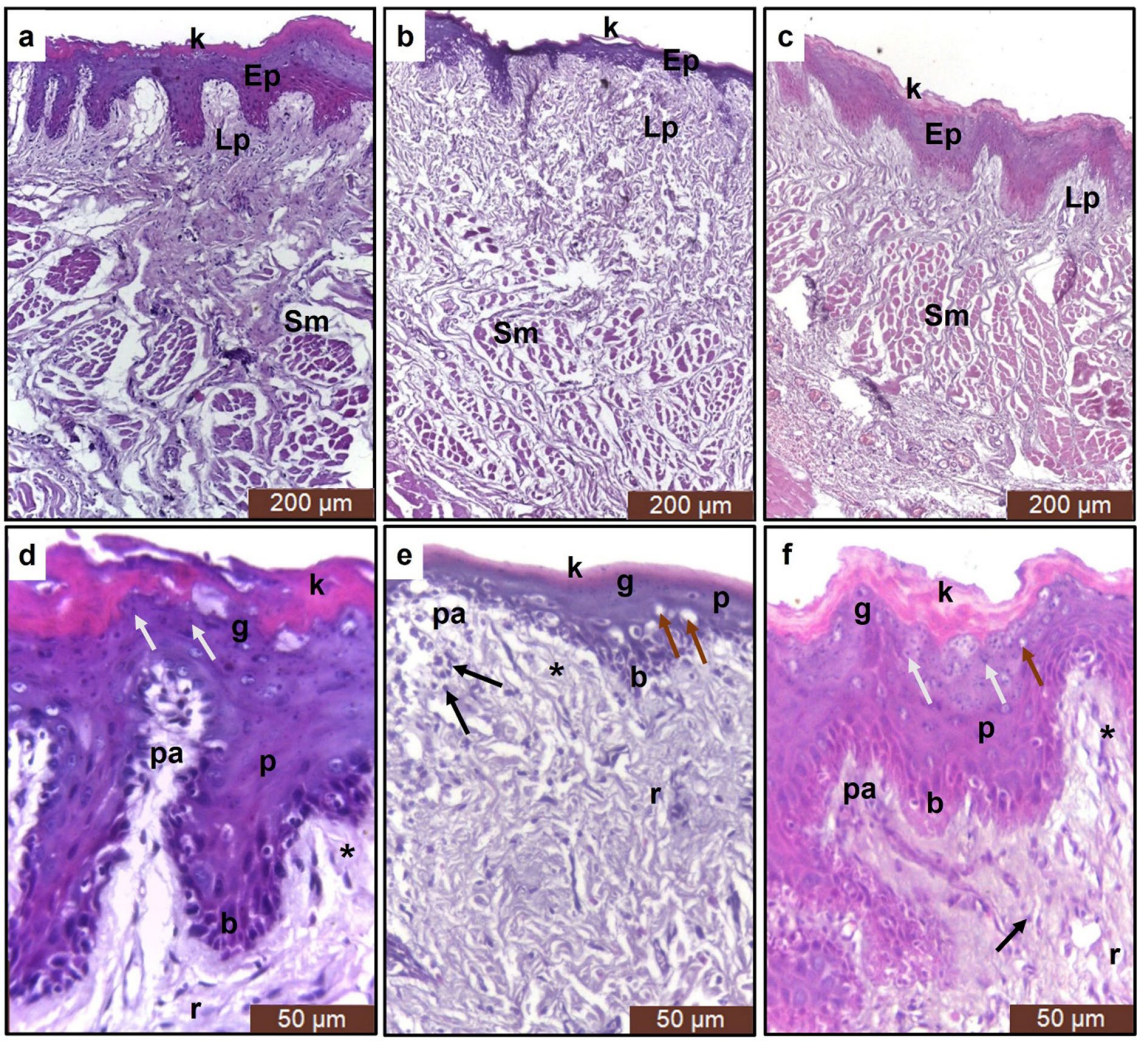
A photomicrograph of the buccal mucosa. **a** & **d**: control group, **b** & **e**: aging model group, **c** & **f**: aspirin group. (k) keratin layer, (Ep) epithelium, (Lp) lamina propria, (Sm) submucosa, (b) basal cell layer, (p) prickle cell layer, (g) granular cell layer, (pa) papillary layer, (r) reticular layer, white arrows: keratohyaline granules, brown arrows: vacuoles, black arrows: inflammatory cells, asterisk: connective tissue fibers [H&E, a-c: ×100 & d-f: ×400].

Histopathological examination of the control group revealed typical buccal mucosa architecture with keratinized squamous epithelium. Epithelial rete pegs were numerous, long, and slender. Basal cells were columnar with large, deeply stained nuclei. Prickle cells occupied most of the epithelial thickness but were smaller in size than basal cells. Granular cells were more flattened and showed basophilic keratohyaline granules. The keratin layer had a remarkable thickness. The lamina propria was separated from the epithelium by a well-defined basement membrane. The papillary layer comprised loose connective tissue (C.T.), while the reticular layer showed dense C.T. with wavy collagen fiber bundles. Beneath the lamina propria, the submucosa showed bundles of muscle fibers representing the buccinator muscle. These bundles were separated by C.T. septa (Fig. 3a and d).

As for the aging model group, this group showed decreased epithelial thickness. Rete pegs became too short (nearly absent), showing a nearly flat epithelium. Basal cells became small. Prickle cells showed intracellular vacuolization. The granular layer showed diminished keratohyaline granules. The keratin layer became thin. The lamina propria showed many differences compared to the control group, where the papillary layer was nearly absent. However, the reticular layer showed increased thickness and density, disoriented collagen fiber bundles, and inflammatory cell infiltration. The submucosa did not differ much (Fig. 3b and e).

The aspirin group showed much improvement compared to the aging model group. The epithelial thickness increased. The rete pegs were long, regular and broad. The basal cells became uniform in size and shape. The prickle cells showed few intracellular vacuoles. Some keratohyaline granules existed in the granular layer but still much less than the control group. The keratin layer thickened. Regarding the lamina propria, its overall thickness was still reduced. However, the papillary layer could be seen with much more density of collagen fibers as compared to the control group. Bundles of collagen fibers became organized in the reticular layer. Moreover, inflammatory cell infiltration was remarkably diminished. The submucosa did not show many differences (Fig. 3c and f).

### Ultrastructural examination results

**Fig. 4:**
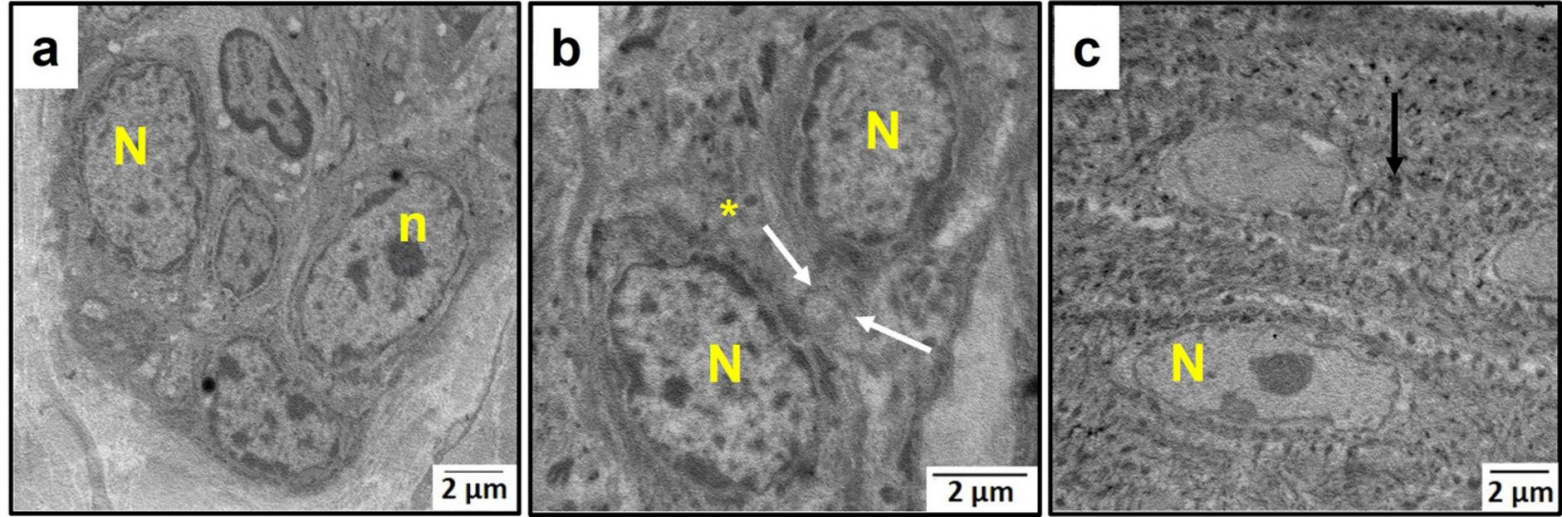
An electron micrograph of the control group’s buccal mucosa showing **a**: basal cells, **b**: prickle cells & **c**: granular cells. (N) nucleus, (n) nucleolus, (asterisk) intercellular spaces, (white arrows) desmosomes, (black arrow) keratohyaline granules. [Magnifications: ×5000, ×8000 & ×5000, respectively].

Ultrastructural examination of the control group revealed typical structure of the buccal mucosal epithelium including basal, prickle, granular, and keratin layers. Normal nuclei of the basal cells were visible, with nucleoli inside them and surrounded by an intact nuclear membrane (Fig. 4a). Prickle cells were connected by desmosomes and showed normal nuclei (Fig. 4b). Granular cells also showed normal nuclear morphology with abundant keratohyaline granules (Fig. 4c).

**Fig. 5:**
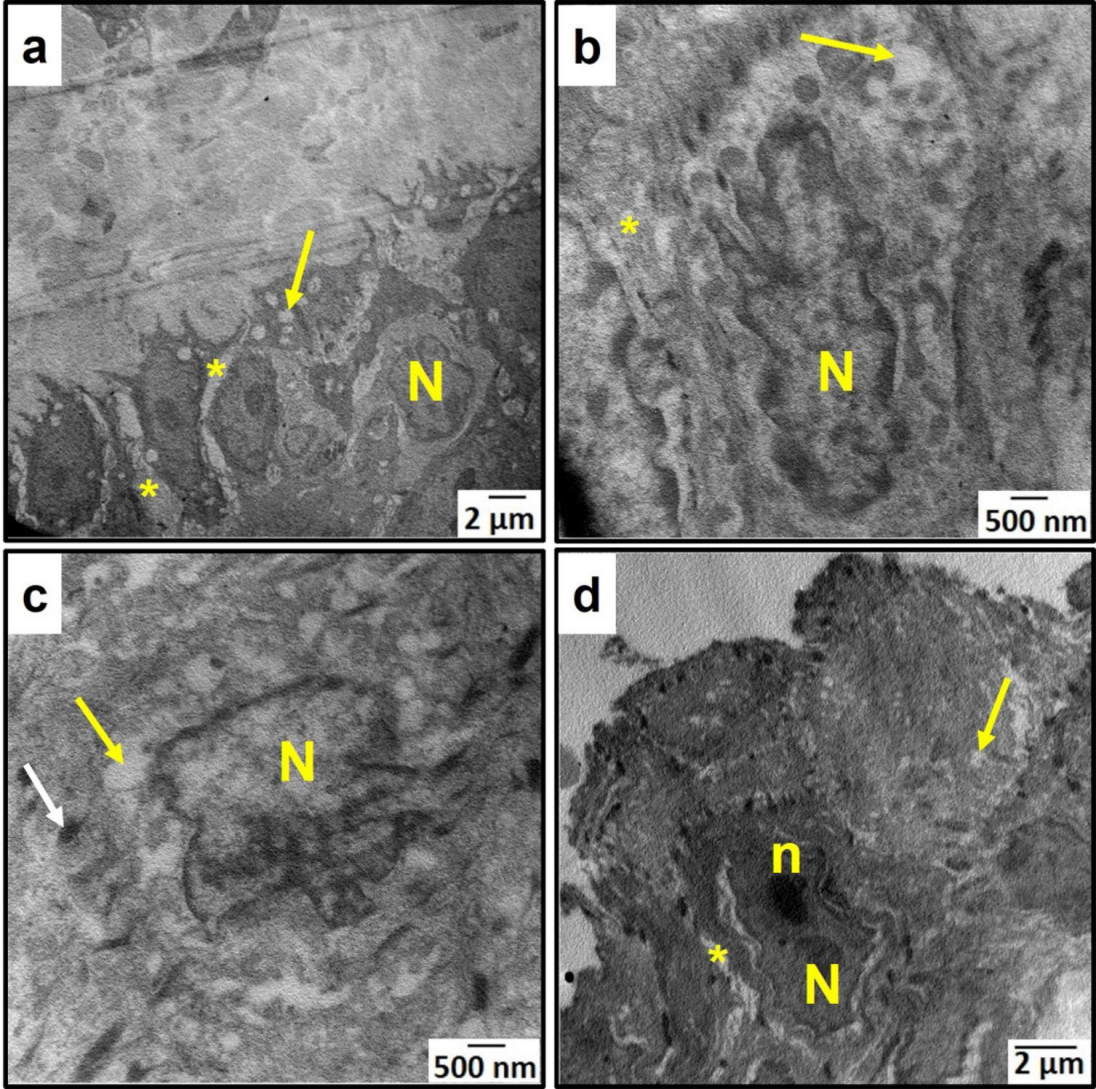
An electron micrograph of the aging model group’s buccal mucosa showing a & b: basal cells. **a**: showing increased intercellular spacing. **b**: showing atypical nuclear morphology. **c**: prickle cells. **d**: granular cells. (N) nucleus, (n) nucleolus, (yellow arrows) vacuoles, (asterisks) intercellular spaces, (white arrow) desmosomes. [Magnifications: ×3000, ×12000, ×12000 & ×5000, respectively].

Regarding the aging model group, keratinocytes of the basal layer showed marked degeneration with increased spacing around them (Fig. 5a). Basal and prickle cells showed many vacuoles intracellularly and intercellularly, and their nuclei showed atypical morphology (Fig. 5b and c). In granular cells, keratohyaline granules were diminished, and vacuoles were also observed (Fig. 5d).

**Fig. 6:**
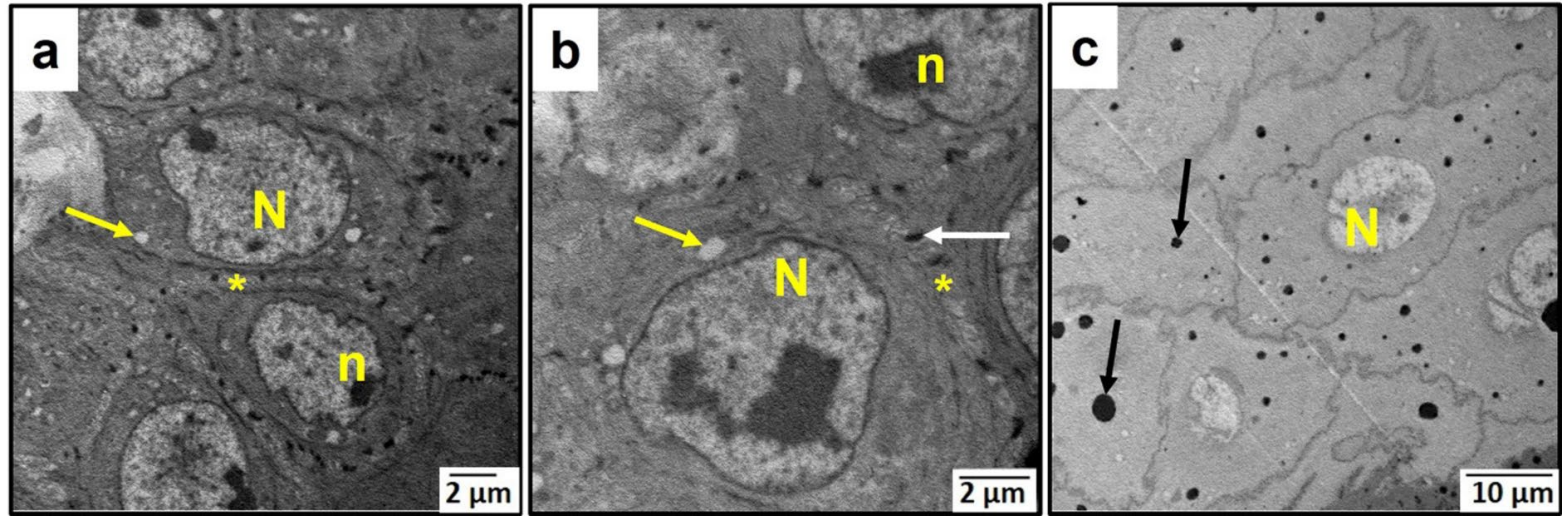
An electron micrograph of the aspirin group’s buccal mucosa showing **a**: basal cells. **b**: prickle cells. **c**: granular cells. (N) nucleus, (n) nucleolus, (yellow arrows) vacuoles, (asterisks) intercellular spaces, (white arrow) desmosomes, (black arrows) keratohyaline granules. [Magnifications: ×4000, ×6000 &×1500, respectively].

As for the aspirin group, keratinocytes of basal and prickle cell layers restored their typical appearance with nearly no signs of degeneration. Spacing decreased around cells and was comparable to the control group. Basal and prickle cells’ nuclei restored their normal morphology. However, there were still some vacuoles (Fig. 6a and b). Regarding the granular cell layer, granular cells showed normal appearance with normal nuclei. In addition, keratohyaline granules were restored, but to a lesser extent when compared to the control group (Fig. 6c).

### Comet analysis

**Fig. 7:**
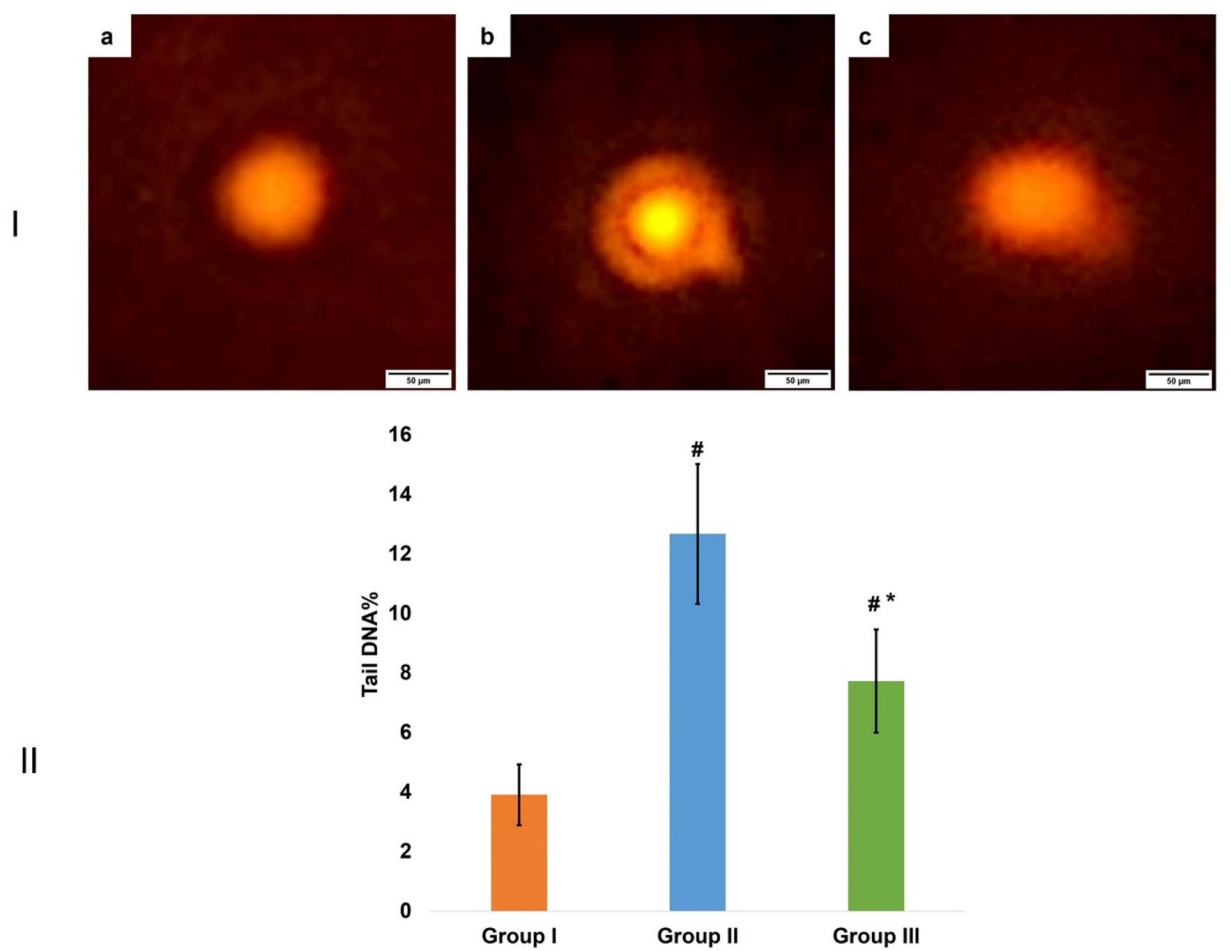
(Ι) DNA damage detected by comet assay. (**a**) control group; (**b**) aging model group; (**c**) aspirin group. (ΙΙ) A bar chart representing the mean ± SD of tail DNA%. # indicates significant difference versus control group. * indicates significant difference versus aging model group. One way ANOVA at p value < 0.05.

The comet assay findings demonstrated no DNA damage in the control group. Treatment with D-galactose markedly enhanced DNA damage as indicated by an increase in tail length. On the other hand, aspirin revealed a dramatic reduction in DNA damage. One-way ANOVA test showed a significant difference in DNA damage between all groups (*P* = 0.001). Regarding pairwise comparison using the post-hoc Tukey test, results showed that the difference between the control and aging model groups was statistically significant (*P* < 0.001). The same applied to the control and aspirin groups (*P* = 0.002) and to the aging model and aspirin groups (*P* < 0.001) (Fig. 7).

## Discussion

There are numerous antiaging strategies, including diet and physical activity, but the most appealing one is reusing already approved drugs by the FDA, such as aspirin, statins, metformin, and rapalogs^4^. Aspirin is an already long studied and effective drug^12^. However, its antiaging mechanism remains ambiguous. Thus, in this study, the antiaging impact of aspirin was studied.

To develop an aging model in the current work, systemic administration of D-galactose was applied^6,13^. Accelerated aging using D-galactose mimics the natural aging process by increasing the production of advanced glycated end products, leading to activation of nuclear factor kappa-light-chain-enhancer of activated B cells (NF-κB), increasing reactive oxygen species (ROS) generation, mitochondrial breakdown, and apoptosis^14,15^.

In the herein study, MDA was selected as a marker for oxidative stress. It was proved by Gil et al. that plasma MDA level increased with aging^16^. This happens as it is produced by ROS reaction with polyunsaturated fatty acids leading to peroxidation of lipids^17^. Lipid peroxidation has significant consequences like loss of proliferation potential of cells, change of gene expression, and suppression of antioxidant enzymes. Consequently, oxidative stress accumulates, and aging occurs^18^. In the current work, the aging model group’s MDA level was higher than the control group. This was in total harmony with prior work performed by Fan et al. and Zhou et al., who showed increased MDA levels in kidneys and brains of mice, respectively, upon D-galactose administration^19,20^.

On the other hand, it was found that aspirin decreased MDA levels compared to the aging model group. This came in harmony with Deng et al., who showed that aspirin provided a shielding effect against endothelial damage caused by low-density lipoprotein (LDL) injection^21^. They found that aspirin inhibited the increase in MDA blood levels caused by LDL. Moreover, in vitro work on endothelial cells cultured in high D-glucose levels showed that aspirin decreased ROS levels significantly^22^. Additionally, aspirin reduced hydrogen peroxide-induced oxidative stress in melanocytes in vitro^23^. This decrease in MDA levels in the aspirin group might be credited to the ability of aspirin to block the metabolism of arachidonic acid into prostaglandins, leading to a decrease in ROS generation that accompanies this process^24^. Moreover, aspirin can induce ferritin synthesis, which has antioxidant properties, thus reducing ROS formation^25^.

In the current work, the iNOS gene was chosen as an oxidative stress and aging marker. This is because iNOS upregulation leads to increased production of nitric oxide, leading to peroxynitrite (ONOO) formation^26^. Increased ONOO formation has severe consequences like suppressing adenosine triphosphate (ATP) synthase, superoxide dismutase, and complexes I and II in the respiratory complex. Additionally, ONOO damages the membranes and DNA of the mitochondria. All this finally leads to oxidative phosphorylation, oxidative stress, and age-dependent reduction in ATP production^27^. In the current work, the aging model group’s iNOS gene expression was greater than the control group. This was concomitant with prior studies in which D-galactose administration in mice caused increased iNOS expression^20,28^.

On the contrary, the aspirin group expressed lower levels of iNOS mRNA expression compared to the aging model group. This result was concomitant with prior work that proved that aspirin suppressed the expression of iNOS in lipopolysaccharide-induced macrophage activation^29^. Moreover, aspirin ameliorated intervertebral disc degeneration induced via percutaneous disc puncture in Sprague-Dawley rats by inhibiting iNOS expression^30^. Aspirin may have decreased iNOS mRNA expression via suppression of NF-κB, which in turn inhibits M1 macrophage polarization. Decreased M1 macrophages ultimately decreases iNOS production^29^. Additionally, aspirin can activate adenosine monophosphate-activated protein kinase, resulting in diminished levels of iNOS, nitric oxide, and matrix metalloproteinases, which finally decrease oxidative damage^30^.

The histological and ultrastructural examination of the buccal mucosa supported all the results mentioned above. The aging model group showed marked degeneration in epithelium and lamina propria of buccal mucosa as compared to the control group. These histological outcomes showed that D-galactose caused marked degeneration in rats’ buccal mucosa. This was similar to Youssef’s results, where aging led to the same degenerative changes in rats’ buccal mucosa^31^. Moreover, Meng et al. demonstrated that gastric mucosa showed marked atrophy after D-galactose injection^32^.

Moving to the ultrastructural analysis, this study showed that D-galactose caused keratinocyte degeneration and atypical nuclear morphology. Similar results were observed ultrastructurally upon normal aging in tongue epithelium of rats^33^. Moreover, Lopes et al. showed concomitant results^34^.

However, in the aspirin-treated group, both histopathological and ultrastructural examinations were appealing, where aspirin protected the buccal mucosa against the deleterious effects of D-galactose. This agreed with Rahman et al.’s study which showed that the skin of C57BL/6 mice exposed to ultraviolet radiation showed decreased inflammatory cell infiltration upon daily oral administration of aspirin^35^. In the herein context, the prophylactic antiaging effect of aspirin on tissues may be accredited to the aspirin’s antioxidant and anti-inflammatory properties. Aspirin can increase antioxidant genes expression like catalase and superoxide dismutase and inhibit NF-κB. Inhibiting NF-κB can decrease iNOS expression and ROS production, causing antioxidant and anti-inflammatory effects and thus preserving the tissues^26,36^.

The comet assay was performed in the current work as it can quantitatively detect DNA breakage and mutations^37^. In the current study, compared to the control group, the aging model group had a higher percentage of DNA breakage (% tail DNA). This was also shown in previous studies, which showed increased DNA breakage in the aging model group in cerebellum cells and immune cells in mice^38,39^.

In the present study, %DNA breakage (% tail DNA) was reduced in the aspirin group as compared to the aging model group. This coincided with a study performed by Dandah, who evaluated how aspirin affected lymphocytes taken from individuals with breast cancer. Using comet assay, they demonstrated that aspirin decreased DNA damage in lymphocytes in vitro^40^. Moreover, another study by Rahman et al. revealed that aspirin protected keratinocytes from ultraviolet radiation-dependent DNA damage by reducing 8-oxoguanine and cyclobutane pyrimidine dimers, markers of oxidative DNA damage, in keratinocytes and melanocytes in skin lesions^35^. Many mechanisms were introduced to explain the aspirin protective effect on DNA, including inhibition of cyclooxygenase enzyme and scavenging of ROS^41^. Furthermore, aspirin could directly inhibit tumor suppressor gene p53, which directly decreases DNA damage and increases cell survival^40^.

Although aspirin is a very promising antiaging drug, benefit risk analysis must be done before it can be recommended. This is due to many reasons, including the inherent side effects of aspirin’s long usage, like bleeding, especially intracerebral hemorrhage, an increasing risk of gastrointestinal tract bleeding, drug-drug interaction with other non-steroidal anti‐inflammatory drugs, or methotrexate^42,43^.

### Limitations

While this study provides preliminary insights into aspirin’s potential antiaging effects, some limitations should be acknowledged. The 8-week duration may be insufficient to capture long-term aging-related changes, and the use of a single dose of aspirin precludes conclusions about dose-dependent efficacy or safety. Furthermore, this study focused on MDA as a biomarker of lipid peroxidation, a well-established indicator of oxidative stress. However, the inclusion of additional markers could provide a more comprehensive assessment of antioxidant activity and redox balance.

## Conclusion

This study demonstrates that aspirin exhibits significant antiaging potential in aged rat models, as evidenced by its ability to attenuate oxidative stress through marked reductions in MDA levels, suppression of iNOS gene expression, and mitigation of DNA damage. Furthermore, histological and ultrastructural analyses revealed substantial improvements in buccal mucosal integrity, underscoring aspirin’s protective role at the tissue and cellular levels. These results lay a critical foundation for future investigations into aspirin’s mechanistic pathways and its broader role in promoting healthy aging.

### Recommendations

While these preclinical findings highlight aspirin’s promise as a therapeutic candidate for age-related oxidative damage, further translational research is warranted to validate its efficacy and safety in human populations. Long-term clinical studies are essential to assess sustained benefits, optimal dosing regimens, and potential side effects, ensuring a comprehensive understanding of aspirin’s applicability in antiaging interventions. Comparative studies with antiaging drugs and synergistic approaches need prioritization.

## Data Availability

All data generated or analyzed during this study are included in this published article.

## Abbreviations

ANOVA: Analysis of variance
ATP: Adenosine triphosphate
C.T.: Connective tissue
FDA: Food and Drug Administration
GAPDH: Glyceraldehyde 3-phosphate dehydrogenase
H&E: Hematoxylin and eosin
iNOS: Inducible nitric oxide synthase
LDL: Low-density lipoprotein
MDA: Malondialdehyde
NF-κB: Nuclear factor kappa-light-chain-enhancer of activated B cells
ONOO: Peroxynitrite
qRT-PCR: Quantitative real-time polymerase chain reaction
ROS: Reactive oxygen species
SD: Standard deviation
